# Persistent Beetroot Colored Urine in a Three‐Year‐Old Child: A Case Report

**DOI:** 10.1002/ccr3.71323

**Published:** 2025-10-15

**Authors:** Pauline Harper, Carl‐Johan Törnhage, Eliane Sardh

**Affiliations:** ^1^ Department of Medical Biochemistry and Biophysics, Centre for Inherited Metabolic Diseases, Porphyria Centre Sweden, Karolinska Institutet Karolinska University Hospital Stockholm Sweden; ^2^ Institute of Clinical Science, Sahlgrenska Academy University of Gothenburg Gothenburg Sweden; ^3^ Department of Pediatrics Skaraborg's Hospital Skovde Sweden; ^4^ Department of Molecular Medicine and Surgery, Centre for Inherited Metabolic Diseases, Porphyria Centre Sweden, Karolinska Institutet Karolinska University Hospital Stockholm Sweden; ^5^ Department of Endocrinology, Karolinska Institutet Karolinska University Hospital Stockholm Sweden

**Keywords:** childhood, dark urine in the diaper, familial porphyria cutanea tarda, hemochromatosis gene carrier, hydroxychloroquine sulfate, phlebotomy

## Abstract

Clinically manifest porphyria cutanea tarda (PCT) is a rare condition in childhood; however, several cases have been reported in the literature and at our centre. Porphyria Center Sweden is a national knowledge centre, and since 1987, we have diagnosed approximately 1400 new cases of manifest PCT, of which only five have been children. All children have been identified as heterozygous carriers of a pathogenic *UROD* gene variant and homozygous for hemochromatosis. In our experience, the diagnosis of children with PCT has been delayed, even in the presence of cutaneous symptoms, without known family history. In this case report, and in another child from our centre, the disease has been suspected due to reddish discolored urine in the absence of cutaneous symptoms. In these two cases, the mothers associated the beetroot red urine with known cases of PCT in the family history. The remaining three cases documented in this report were diagnosed based on cutaneous symptoms.

## Graphical abstract

Clinically manifest porphyria cutanea tarda (PCT) is rare in children. Of ~1400 cases diagnosed at Porphyria Center Sweden, only five were children, all with pathogenic *UROD* variants and *HFE* homozygous. Diagnosis is often delayed; in our case, beetroot‐red urine prompted suspicion despite the absence of cutaneous symptoms.

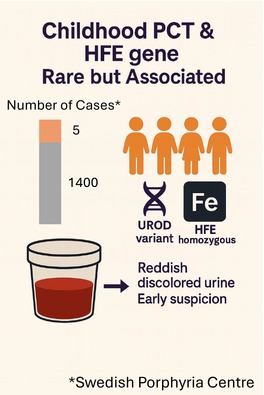

**Summary**

- Symptomatic porphyria cutanea tarda in children is extremely rare and is often overlooked by the pediatrician if the family is unaware of the genetic predisposition.
- Carriers of pathogenic variants in the *UROD* gene should be encouraged to test their children or be alert for reddish‐discolored urine or skin blistering.

## 1 Introduction

**FIGURE 1:**
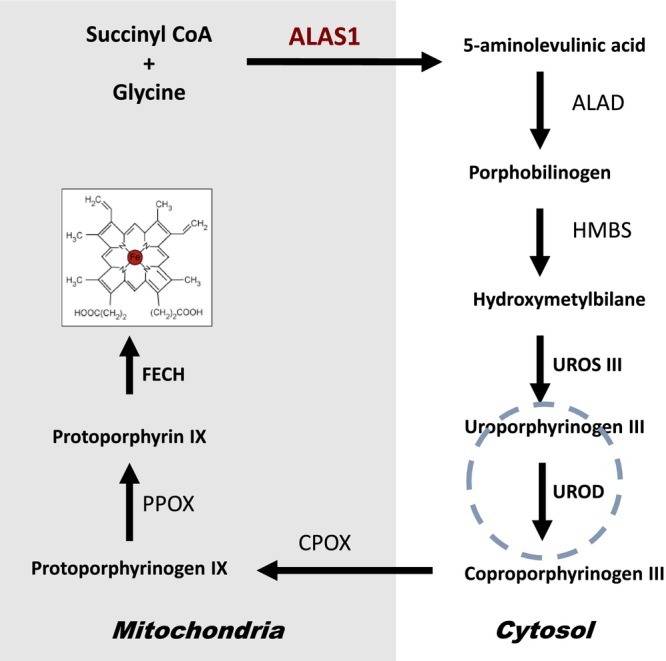
Heme biosynthesis includes eight catalytic steps. The first catalytic step of heme biosynthesis mediated by 5‐aminolevulinic acid synthase (ALAS) takes place in the mitochondrial build of 5‐aminolevulinic acid (ALA). There are two isoforms of ALAS: an erythroid‐specific variant (ALAS2) and a ubiquitously expressed form (ALAS1), which is primarily active in hepatic heme biosynthesis. The subsequent four intermediate steps take place in the cytosol. Two molecules of ALA are combined to form porphobilinogen (PBG). Four PBG molecules are then merged to form hydroxymethylbilane by the third enzyme in the pathway, porphobilinogen deaminase (PBGD). Hydroxymethylbilane is enzymatically converted to a cyclic porphyrin ring, uroporphyrinogen III, which in the fifth catalytic step is mediated by uroporphyrinogen decarboxylase (UROD) and undergoes a series of decarboxylations to form coproporphyrinogen III. The next three synthetic steps take place inside the mitochondrion to form protoporphyrin IX, and the incorporation of ferrous iron into protoporphyrin IX by ferrochelatase (FECH) results in the heme molecule.

Porphyria cutanea tarda (PCT) is caused by a significantly reduced activity of the liver enzyme uroporphyrinogen decarboxylase [1] (UROD, Figure 1). The sporadic form of PCT is the most prevalent, accounting for 80% of cases without a known genetic basis. In contrast, the familial type of PCT is genetically inherited as an autosomal dominant trait with pathogenic variants in the *UROD* gene. The reduced activity of the UROD enzyme leads to the accumulation of phototoxic porphyrins, mainly uroporphyrinogen and heptacarboxylated porphyrinogens that cause the characteristic clinical pattern of skin fragility and fluid‐filled blistering on sun‐exposed skin areas. Iron plays a central role in the pathogenesis of PCT, causing an important inhibition of UROD enzyme activity, which is reversible by treatment. Patients with sporadic PCT typically become symptomatic around the 5th–6th decade, but the genetic form may occur earlier, at age 40–50 years [2]. The symptoms are most common in summer and autumn months. The urine of a patient with PCT is often brown‐red colored when exposed to air or light due to the high concentration of porphyrins. Pediatric cases are rare [3], sharing the same pathophysiology and cutaneous manifestations as adults.

## 2 Case History

The boy was born prematurely at 25 + 6 weeks gestation in an uneventful twin pregnancy, but due to preterm labor, an acute cesarean section was performed. The infant weighed 730 g at birth and required highly specialized neonatal care. Immediately after birth, he received surfactant prophylaxis, intravenous antibiotics, and assisted ventilation for 4 days. Nutritional support was provided by administering a combination of breast milk (1/3 of the total volume) and breast milk substitute (Neocate, 2/3 of the total volume) at a rate of 150–200 mL/kg body weight per day. This nutritional regimen continued until the infant reached three and a half months of age. Thereafter, the infant was given 900 mL breast milk substitute daily, and other types of child‐adapted food, including oatmeal, fruit smoothies, and porridge, were gradually introduced. From the age of 13 months onwards, the infant was introduced to solid foods and ate with a good appetite. He received iron supplementation (30 mg/mL Niferex), 0.1 mL × 2 (1.5 mg/kg) according to national guidelines for age and weight from one to 6 months of age. He continued iron supplementation at 30 mg/mL, 3 drips daily, 4.5 mg (0.8 mg/kg weight) until one year of age. He was discharged from neonatal care in good health at the age of 41 weeks (9.4 months) with a weight of 5840 g. Subsequent medical examinations showed good growth and normal psychomotor development.

**FIGURE 2:**
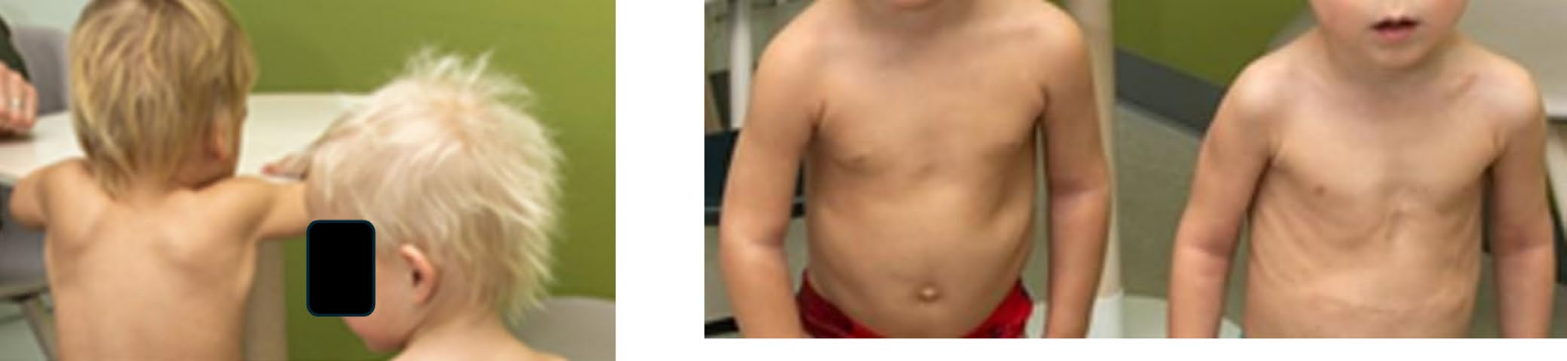
At two years of age, the twin brothers display a notable difference in pigmentation. The index case (left) exhibits visibly darker hair and skin compared to his twin brother (right).

At a follow‐up visit at the age of 2½ years, the boy weighed 14.9 kg and was 96.6 cm tall. The skin appeared hyperpigmented and darker hair, compared to the skin and hair of his twin brother (Figure 2) [4] and had some eczematous lesions. The parents reported that the boy had a long history of dark urine with a beetroot or brown color. On presentation, the boy was pain‐free, the physical examination was normal, and he had no signs of infection or fever. The parents denied ingesting any food containing beetroot or other red pigments. Family history revealed that the mother had a paternal uncle with hereditary PCT, and she wondered if this might be the cause of her son's beetroot‐colored urine. With this question, the attending physician contacted the Porphyria Center Sweden, who strongly recommended an investigation of porphyria.

## 3 Methods: Porphyria Investigation and Differential Diagnosis

A laboratory investigation was also performed to rule out other causes of reddish discoloration of urine and skin pigmentation. Urinalysis was normal with no evidence of hematuria, myoglobinuria, or urinary tract infection. Blood hemoglobin was normal at 133 g/L (normal range: 100–150). Liver function tests were mildly elevated; plasma aspartate aminotransaminase (AST) 1.2 μkat/L (normal range: 0.41–0.93), plasma alanine aminotransaminase (ALT) 1.4 μkat/L (normal range: 0.19–0.51). Plasma bilirubin 6 μmol/L, plasma gamma‐glutamyltransferase, and pancreatic amylase were all normal. Other causes of hyperpigmentation, such as Addison's disease, were ruled out with normal plasma adrenocorticotropic hormone 3.3 pmol/L (normal range: 2–11), plasma 17‐hydroxyprogesterone 0.22 nmol/L (< 2.7), and serum cortisol 368 nmol/L (normal range: 145–620). Abdominal ultrasound showed normal findings including liver, spleen, and kidneys. There was no evidence of intrahepatic cholestasis, and the gallbladder was normal without gallstones.

PCT investigation was started in winter, at the end of December 2022. Plasma fluorescence emission showed a distinct peak at 617 nm, with the likely diagnosis of PCT. Further investigation revealed elevated plasma and urinary porphyrins: 182.3 nmol/L (reference ≤ 10) and 1115 μmol/mol crea (reference ≤ 38), respectively, with dominance of polycarboxylated porphyrins in urine, confirming the diagnosis of PCT. Two weeks later, with an additional control sample taken before treatment, the biochemical pattern had worsened: p‐porphyrins 401.2 nmol/L and u‐porphyrins 2571 μmol/mol crea, indicating a long‐standing inhibition of hepatic UROD. Urinary 5‐aminolevulinic acid (ALA) and porphobilinogen (PBG) were elevated (9.9 mmol/mol crea, reference ≤ 2.0, and 1.3 mmol/mol crea, reference ≤ 0.13, respectively), which are rarely increased in PCT, indicating a stressed hepatic heme biosynthetic state (Figure 1).

**FIGURE 3:**
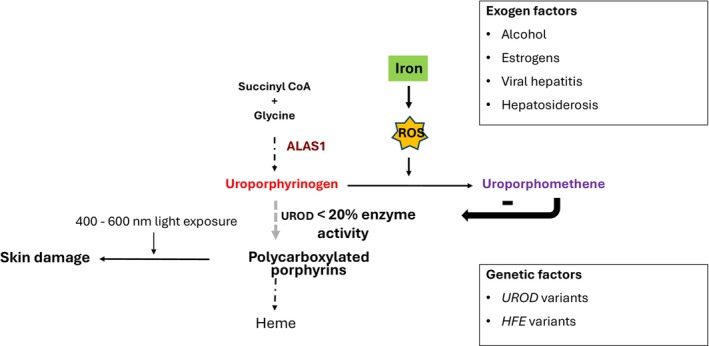
The primary metabolic defect PCT is a deficiency of hepatic UROD. Inhibition of this enzyme, whether through acquired deficiency of activity (sporadic PCT) or hereditary partial deficiency (familial PCT), is caused by additional factors reducing the enzyme activity below 20% of normal, resulting in the accumulation of polycarboxylated porphyrins damaging the skin. The major additional factors that diminish the activity of UROD include alcohol‐related liver disease, chronic viral hepatitis, exposure to estrogens, and iron overload (usually related to *HFE* variants). Accumulated uroporphyrinogen in the cytosol is partially oxidized to uroporphomethene in the presence of iron and other oxidative stressors. Uroporphomethene can act as a UROD inhibitor. Reactive Oxygen Species (ROS). The key diagnostic feature is a marked increase in urinary porphyrins, with predominance of uro‐ and heptacarboxyl porphyrins (polycarboxylated porphyrins).

DNA sequencing of the *UROD* variant present in the family identified the child to be a heterozygous carrier of the pathogenic variant c.651dupT; p.Glu218, which introduces an early stop codon and leads to truncation of the protein [5]. Given the strong association between predisposition to hemochromatosis in children with PCT, the boy was genetically tested for the two major *HFE* variants (C282Y; c.845G>A and H63D; c.187C>G). The child was found to be homozygous for the *HFE* gene variant c.845G>A; p.C282Y [6]. No other possible triggers for overt PCT were found [7] (Figure 3).

## 4 Treatment Outcome and Follow‐Up

**FIGURE 4:**
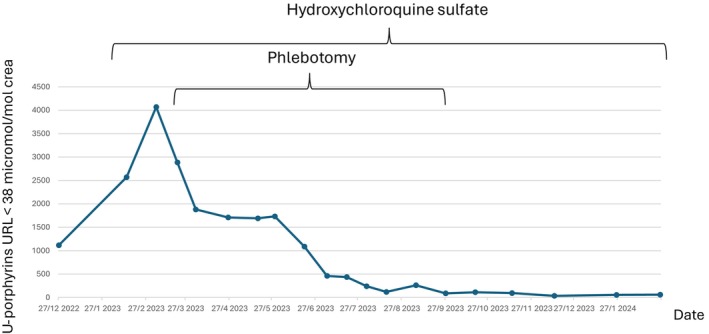
Urinary porphyrins at diagnosis and during treatment. The patient reached biochemical remission after 9 months of treatment.

**FIGURE 5:**
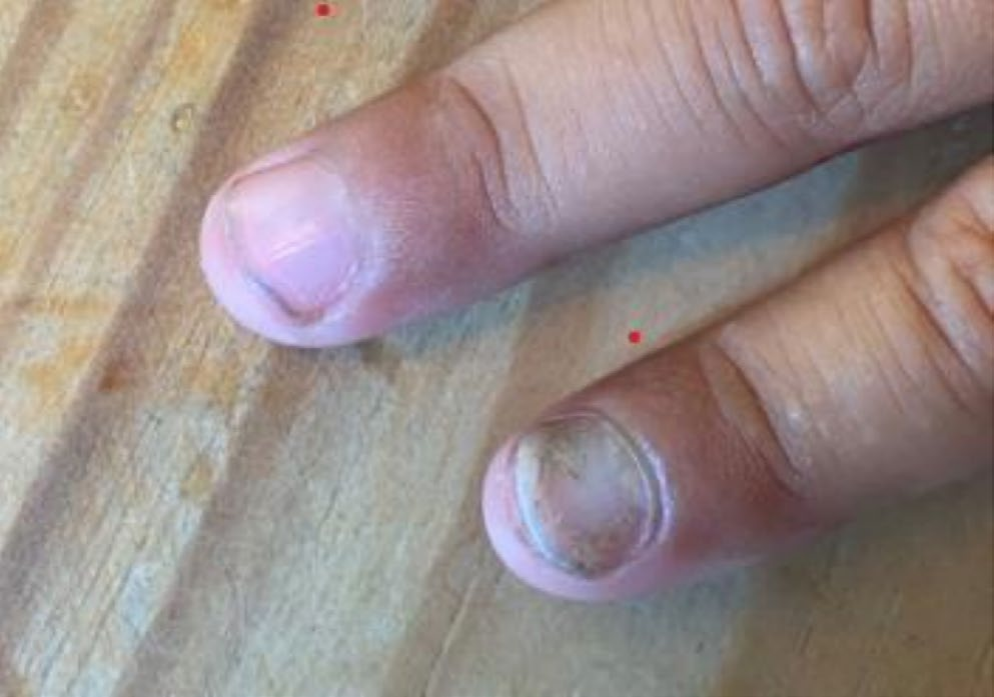
Porphyria cutanea tarda (PCT) presents clinically with blistering lesions in sun‐exposed areas of the skin. A frequently associated manifestation is onycholysis, with typical distal separation of the nail plate from the underlying nail bed, and a white opaque region on the affected nail (bottom).

The treating pediatrician started the boy on hydroxychloroquine sulfate (Plaquenil) 45 mg twice a week 40 days after diagnosis, after an ophthalmological examination, which is recommended before starting hydroxychloroquine sulfate treatment. This was even more important in this case because the boy had Grade 2–3 retinopathy of prematurity and was treated with laser therapy 3 months after birth. On examination, visual acuity was 1.0 in the right eye and 0.9 in the left. Optical coherence tomography showed a normal retina. Following the introduction of hydroxychloroquine sulfate, urinary porphyrins levels increased (Figure 4), as did plasma liver transaminase levels (AST 3.0 μkat/L and ALT 4.2 μkat/L), and a series of 16 therapeutic phlebotomies was started 60 days after diagnosis, with 90 mL of blood (6 mL/kg body weight) drawn every 2–3 weeks, maintaining an age‐appropriate B‐Hb level. Levels of ALA and PBG slowly returned to normal levels four months after the start of treatment. During the first summer after diagnosis, the child showed cutaneous symptoms in the form of onycholysis (Figure 5) and small blisters on sun exposure.

Except for this brief symptomatic skin period, the child achieved remission within a year on this regimen and has been in remission ever since. A maintenance dose of hydroxychloroquine sulfate twice weekly was continued during the following summer and then discontinued. Figure 4 summarizes the urinary porphyrins correlated with treatment modality.

## 5 Therapy and Iron Homeostasis

Treatment was initiated with hydroxychloroquine sulfate, which enhances the water solubility of porphyrins accumulated in the liver, thereby promoting their urinary excretion. Concurrently, phlebotomy was planned, with careful consideration of the appropriate blood volume and the need for venous access in a three‐year‐old child. The purpose of phlebotomy was to remove iron from the body and enhance the mobilization of iron from hepatic stores to erythropoietic tissues, thereby relieving iron‐mediated inhibition of hepatic UROD activity. Treatment commenced 60 days after the confirmed diagnosis. The combination of hydroxychloroquine and phlebotomy resulted in significant improvements in both biochemical and clinical outcomes [8, 9].

During the first year of life, the infant received approximately 5000 mg of iron, including iron supplements, breast milk, and breast milk substitutes. Approximately 10%–15% of iron supplementation is absorbed [10]. In patients with hereditary hemochromatosis type 1, duodenal iron absorption is approximately 2–3 times higher than normal [11]. Thus, the child may have absorbed approximately 1500–2000 mg of iron during the first year of life.

At the time of PCT diagnosis, blood hemoglobin (B‐Hb) was 133 g/L (reference range: 100–150 g/L). Sixty days later, and prior to the initiation of venesection treatment, B‐Hb had increased to 143 g/L. Red blood cell porphyrin analysis showed a concentration of 3.3 μmol/L (reference ≤ 1.2 μmol/L), with 95% consisting of zinc protoporphyrin, indicating no iron overload. A T2 MRI scan was performed after approximately three phlebotomies, revealing no evidence of iron overload in the heart, liver, or pancreas.

A total of 16 phlebotomies were performed over a nine‐month period, during which 1440 mL of blood was removed, representing a total iron loss of approximately 720 mg. During this period, fifteen B‐Hb measurements were taken, with a mean value of 128 g/L, ranging from 146 g/L at baseline to 111 g/L at the end of treatment. At diagnosis, plasma iron was 28 μmol/L (reference range: 5–28 μmol/L), and plasma ferritin was 121 μg/L (reference range: 13–300 μg/L). Following the initiation of phlebotomy, plasma iron levels gradually decreased to 6–9 μmol/L after nine months of treatment. Plasma ferritin increased to 401 μg/L around the same time that plasma AST and ALT levels peaked, just before the start of therapeutic phlebotomies, to then decline to < 10 μg/L after nine months of treatment. Total iron‐binding capacity remained stable throughout the treatment period.

## 6 Discussion

Porphyria cutanea tarda is characterized by skin fragility and blistering in sun‐exposed areas and is commonly associated with liver dysfunction and iron overload [2]. The term *porphyria cutanea tarda* was coined by Jan Waldenström in 1937, noting its typically late onset (“tarda”) and later described its familial form in 1963 [12]. The debate about whether PCT was inherited or acquired was resolved in 1976 by de Verneuil and colleagues, who showed that PCT can be either sporadic or familial—the latter caused by an autosomal dominant *UROD* mutation [13]. Familial PCT accounts for about 20% of cases [2, 14]. In 1985, Elder et al. [15] demonstrated that hepatic UROD activity is reduced in all active PCT cases, regardless of type, but the activity returned to baseline during phlebotomy‐induced remission. This suggests that hepatic UROD activity is inhibited during manifest PCT, and that this inhibition correlated with hepatic iron stores. Although most PCT patients have elevated iron stores (rarely at hemochromatosis levels), iron depletion is effective even when levels are within the normal range [16]. Relapse can occur after iron repletion [17]. In 2007, Phillips et al. [18] identified *uroporphomethene*, an iron‐dependent inhibitor of UROD, reinforcing the link between iron and disease activity (Figure 3). Another treatment modality arose from the observation that antimalarial doses of chloroquine can cause a rapid release of liver porphyrins that reduce photosensitivity [19]. Long‐term administration of a lower dose has proved to be an effective treatment, increasing the excretion of porphyrins without causing significant liver damage [2].

The first reported cases of children affected by PCT were those in eastern Turkey caused by bread made from wheat seeds treated with the fungicide hexachlorobenzene (HCB) [20]. Hexachlorobenzene or a metabolite acts directly in experimental animals by decreasing the activity of UROD specifically in the liver [21].

In the following years, several cases of children developing overt PCT were reported. All these reports have similarities to the cases we have seen in our cohort. Although in most cases the child has developed skin blisters at the time of diagnosis [3, 8, 9, 22, 23] in contrast to our case which showed no skin manifestations at diagnosis. These cases describe the onset of skin fragility in children between the ages of 2 and 7 years, affecting both girls and boys. Clinical features included skin fragility, vesicles, scarring, and milia, primarily on the hands and facial skin. Hypertrichosis was noted in several cases [3]. Most of the children were found to have family members with decreased UROD enzyme activity, suggesting hereditary PCT [22]. Mild abnormalities in liver function tests or slightly elevated iron indices were observed in a few cases. In these cases, no specific triggering factor other than a presumed hereditary cause of PCT has been reported. The described treatments included age‐ and weight‐adjusted phlebotomies [8] or low‐dose hydroxychloroquine administered twice weekly [9], both of which led to good clinical responses. In two cases, symptom onset was suspected to be drug‐induced (griseofulvin, hydantoins), indicating possible toxin‐triggered PCT [23].

The child in this report, as well as the other children in our cohort shown in Table 1 [6], is a carrier of a pathogenic *UROD* variant and a homozygous carrier of hereditary hemochromatosis type 1. *HFE* gene mutations are common in people of Northern European ancestry, and the cause of limited penetrance is unclear [24]. Symptomatic hereditary hemochromatosis rarely occurs before the age of 30 [25]. The frequency of pathogenic *UROD* variants in the general population is unknown.

**TABLE 1:** In Sweden, only four children have so far been diagnosed with clinical PCT before puberty.

	Case A	Case B	Case C	Case D
Age at onset of symptoms	18 months	5 years	5 years	11 years
U‐porphyrins, micromol/mol crea (URL < 38)	1.146	262	540	387
Treatment	Discontinued hepatotoxic treatment	Phlebotomy	Phlebotomy	Phlebotomy and chloroquine phosphate
*UROD* variant	636+1G>C	876‐7_878dup10bp	876‐7_878dup10bp	876‐7_878dup10bp
*HFE* variant homozygous	c.845G>A (C282Y)	c.845G>A (C282Y)	c.187C>G (H63D)	c.187C>G (H63D)
Known family history	Yes	No	No	Yes

*Note:* All are girls with genetic PCT and *HFE* homozygous. Cases A and D were diagnosed in connection with the onset of symptoms; cases B and C were symptomatic in early childhood but were diagnosed only after starting anticontraceptive treatment.

Abbreviations: *HFE*, Hereditary Hemochromatosis Gene; PCT, Porphyria Cutanea Tarda; *UROD*, Uroporphyrinogen Decarboxylase Gene.

The fact that all children with PCT in our cohort have been diagnosed with hereditary hemochromatosis type 1 suggests that increased iron absorption in combination with a heterozygous *UROD* variant may contribute to the early onset of clinically active PCT. Case A in Table 1 was diagnosed at 18 months of age due to extremely brown‐colored urine. Treatment with valproic acid for epilepsy likely triggered increased urinary porphyrin excretion. The diagnosis was suspected by the mother, who had previously been diagnosed with familial PCT. Case B experienced skin blistering from the age of five and was finally correctly diagnosed and treated at the age of 13. Case C had a long‐standing history of reddish‐brown urine and blistering, later developing facial hypertrichosis. She showed significant clinical improvement after menarche, but symptoms reappeared upon initiation of oral contraceptives, at which point the diagnosis was confirmed and treatment initiated. Case D developed skin lesions and blisters at age 11. The diagnosis was established promptly due to a grandparent's history of familial PCT and prior genetic counseling for the family [26].

## 7 Conclusion

Based on previous case reports and our own experience, this case highlights the genetic contribution to early‐onset PCT, in contrast to late‐onset PCT where environmental factors play a major role in triggering symptoms. The main message is that when an individual is identified as a heterozygous carrier of a pathogenic *UROD* variant, consultation with a medical geneticist or certified genetic counselor should be arranged. This ensures that affected individuals and their families are appropriately informed about the significance of the finding, the inheritance pattern, and the implications of familial PCT, thereby supporting informed medical and personal decision‐making. It may also be advisable to investigate the presence of pathogenic *HFE* variants that contribute to iron overload.

## Author Contributions

**Pauline Harper:** conceptualization, data curation, formal analysis, supervision, validation, writing – original draft, writing – review and editing. **Carl‐Johan Törnhage:** conceptualization, writing – review and editing. **Eliane Sardh:** conceptualization, data curation, formal analysis, supervision, validation, writing – review and editing.

## Ethics Statement

The guardians provided written informed consent to allow for their child's de‐identified medical and laboratory information to be used in this publication.

## Consent

Written informed consent was obtained from the patient guardians to publish this case report in accordance with the journal's patient consent policy.

## Conflicts of Interest

The authors declare no conflicts of interest.

## Data Availability

Swedish porphyria registry (a national registry managed by the Health Care Board in Region Stockholm and approved by the Swedish Data Protection Authority (No. 647‐88)). The data is not publicly available due to restrictions on privacy and ethics.

## References

[1] J. D. Phillips , “Heme Biosynthesis and the Porphyrias,” Molecular Genetics and Metabolism 128, no. 3 (2019): 164–177, 10.1016/j.ymgme.2019.04.008. 31326287 PMC7252266

[2] A. K. Singal , “Porphyria Cutanea Tarda: Recent Update,” Molecular Genetics and Metabolism 128, no. 3 (2019): 271–281, 10.1016/j.ymgme.2019.01.004. 30683557

[3] G. H. Elder , “Hepatic Porphyrias in Children,” Journal of Inherited Metabolic Disease 20, no. 2 (1997): 237–246, 10.1023/a:1005313024076. 9211196

[4] F. C. Shaffrali , A. J. McDonagh , and A. G. Messenger , “Hair Darkening in Porphyria Cutanea Tarda,” British Journal of Dermatology 146, no. 2 (2002): 325–329, 10.1046/j.1365-2133.2002.04591.x. 11903250

[5] J. D. Phillips , T. L. Parker , H. L. Schubert , F. G. Whitby , C. P. Hill , and J. P. Kushner , “Functional Consequences of Naturally Occurring Mutations in Human Uroporphyrinogen Decarboxylase,” Blood 98, no. 12 (2001): 3179–3185, 10.1182/blood.v98.12.3179. 11719352

[6] P. Harper , Y. Floderus , P. Holmström , G. Eggertsen , and M. Gåfvels , “Enrichment of HFE Mutations in Swedish Patients With Familial and Sporadic Form of Porphyria Cutanea Tarda,” Journal of Internal Medicine 255, no. 6 (2004): 684–687, 10.1111/j.1365-2796.2004.01309.x. 15147533

[7] S. Thunell and P. Harper , “Porphyrins, Porphyrin Metabolism, Porphyrias. III. Diagnosis, Care and Monitoring in Porphyria Cutanea Tarda–Suggestions for a Handling Programme,” Scandinavian Journal of Clinical and Laboratory Investigation 60, no. 7 (2000): 561–579. 11202050

[8] M. B. Poh‐Fitzpatrick , P. J. Honig , H. C. Kim , and S. Sassa , “Childhood‐Onset Familial Porphyria Cutanea Tarda: Effects of Therapeutic Phlebotomy,” Journal of the American Academy of Dermatology 27 (1992): 896–900, 10.1016/0190-9622(92)70277-m. 1361499

[9] A. J. Bruce and I. Ahmed , “Childhood‐Onset Porphyria Cutanea Tarda: Successful Therapy With Low‐Dose Hydroxychloroquine (Plaquenil),” Journal of the American Academy of Dermatology 38, no. 5 Pt 2 (1998): 810–814, 10.1016/s0190-9622(98)70464-5. 9591792

[10] K. Pantopoulos , “Oral Iron Supplementation: New Formulations, Old Questions,” Haematologica 109, no. 9 (2024): 2790–2801, 10.3324/haematol.2024.284967. 38618666 PMC11367235

[11] P. Brissot , M. B. Troadec , and O. Loreal , “Intestinal Absorption of Iron in HFE‐1 Hemochromatosis: Local or Systemic Process?,” Journal of Hepatology 40, no. 4 (2004): 702–709, 10.1016/j.jhep.2004.01.020. 15030990

[12] J. Waldenström and B. Haeger‐Aronsen , “Different Patterns of Human Porphyria,” British Medical Journal 3 (1963): 272–276, 10.1136/bmj.2.5352.272. PMC1872399 13998428

[13] H. de Verneuil , G. Aitken , and Y. Nordmann , “Familial and Sporadic Porphyria Cutanea. Two Different Diseases,” Human Genetics 44 (1978): 145–151, 10.1007/BF00295407. 730158

[14] G. H. Elder , “Update on Enzyme and Molecular Defects in Porphyria,” Photodermatology, Photoimmunology & Photomedicine 14, no. 2 (1998): 66–69. 10.1111/j.1600-0781.1998.tb00014.x 9638727

[15] G. H. Elder , A. J. Urquhart , R. E. De Salamanca , J. J. Munoz , and H. L. Bonkovsky , “Immunoreactive Uroporphyrinogen Decarboxylase in the Liver in Porphyria Cutanea Tarda,” Lancet II (1985): 229–233, 10.1016/s0140-6736(85)90287-9. 2862415

[16] O. Lundvall , A. Weinfeld , and P. Lundin , “Iron Storage in Porphyria Cutanea Tarda,” Acta Medica Scandinavica 1–2, no. 1 (1970): 37–53, 10.1111/j.0954-6820.1970.tb08003.x. 5507243

[17] O. Lundvall and A. Weinfeld , “Studies of the Clinical and Metabolic Effects of Phlebotomy Treatment in Porphyria Cutanea Tarda,” Acta Medica Scandinavica 184 (1968): 191–199, 10.1111/j.0954-6820.1968.tb02443.x. 5703974

[18] J. D. Phillips , H. A. Bergonia , C. A. Reilly , M. R. Franklin , and J. P. Kushner , “A Porphomethene Inhibitor of Uroporphyrinogen Decarboxylase Causes Porphyria Cutanea Tarda,” Proceedings of the National Academy of Sciences of the United States of America 104, no. 12 (2007): 5079–5084, 10.1073/pnas.0700547104. 17360334 PMC1820519

[19] I. D. London , “Porphyria Cutanea Tarda. Report of a Case Successfully Treated With Chloroquine,” Archives of Dermatology 57 (1957): 801–803, 10.1001/archderm.1957.01550180015004. 13423883

[20] H. A. Peters , A. Gocmen , D. J. Cripps , G. T. Bryan , and I. Dogramaci , “Epidemiology of Hexachlorobenzene‐Induced Porphyria in Turkey. Clinical and Laboratory Follow‐Up After 25 Years,” Archives of Neurology 39 (1982): 744–749, 10.1001/archneur.1982.00510240006002. 7138315

[21] G. H. Elder , J. O. Evans , and S. A. Matlin , “The Effect of the Porphyrogenic Compound, Hexachlorobenzene, on the Activity of Hepatic Uroporphyrinogen Decarboxylase in the Rat,” Clinical Science and Molecular Medicine 51 (1976): 71–80, 10.1042/cs0510071. 939068

[22] M. J. Cruces Prado , R. Enríquez de Salamanca , M. Verea Hernando , M. L. Peña Payero , T. Catalan Beltran , and A. Robledo Aguilar , “Two Cases of Infantile and Familial Porphyria Cutanea Tarda,” Dermatologica 161 (1980): 205–210, 10.1159/000250359. 7398998

[23] J. M. Mascaro , “Porphyrias in Children,” Pediatric Dermatology 9 (1992): 371–372, 10.1111/j.1525-1470.1992.tb00634.x. 1362814

[24] W. C. Palmer , P. Vishnu , W. Sanchez , et al., “Diagnosis and Management of Genetic Iron Overload Disorders,” Journal of General Internal Medicine 33, no. 12 (2018): 2230–2236, 10.1007/s11606-018-4669-2. 30225768 PMC6258594

[25] L. Hashemi and R. Nisenbaum , “A Case‐Based Review of Iron Overload With an Emphasis on Porphyria Cutanea Tarda, Hepatitis C, C282Y Heterozygosity, and Coronary Artery Disease,” Federal Practitioner 37, no. 2 (2020): 95–100. PubMed PMID: 32269472; PubMed Central PMCID: PMC7138343 interest with regard to the article. 32269472 PMC7138343

[26] S. Rudnick , J. Phillips , H. Bonkovsky , and Porphyrias Consortium of the Rare Diseases Clinical Research N , “Familial Porphyria Cutanea Tarda,” in *GeneReviews®*, ed. M. P. Adam, J. Feldman and G. M. Mirzaa, et al. Created: June 6, 2013; Updated: June 9, 2022 (Seattle (WA): University of Washington, Seattle; 1993–2024.: U.S. National Library of Medicine, 2022). 23741761

